# Nimbolide B and Nimbic Acid B, Phytotoxic Substances in Neem Leaves with Allelopathic Activity

**DOI:** 10.3390/molecules19066929

**Published:** 2014-05-26

**Authors:** Hisashi Kato-Noguchi, Md Abdus Salam, Osamu Ohno, Kiyotake Suenaga

**Affiliations:** 1 Department of Applied Biological Science, Faculty of Agriculture, Kagawa University, Miki, Kagawa 761-0795, Japan; E-Mail: salamma71@yahoo.com; 2 Department of Chemistry, Faculty of Science and Technology, Keio University, 3-14-1 Hiyoshi, Kohoku, Yokohama 223-8522, Japan; E-Mails: ohno@chem.keio.ac.jp (O.O.); suenaga@chem.keio.ac.jp (K.S.)

**Keywords:** allelopathy, *Azadirachta indica*, bioactive compound, growth inhibitor, medicinal plant, phytotoxicity

## Abstract

Neem (*Azadirachta indica*) has been widely used as a traditional medicine and several bioactive compounds have been isolated from this species, but to date no potent allelopathic active substance has been reported. Therefore, we investigated possible allelopathic property and phytotoxic substances with allelopathic activity in neem. An aqueous methanol extract of neem leaves inhibited the growth of roots and shoots of cress, lettuce, alfalfa, timothy, crabgrass, ryegrass, barnyard grass and jungle rice. The extracts were then purified by several chromatographic runs while monitoring the inhibitory activity and two phytotoxic substances were isolated. The chemical structures of the two substances were determined by spectral data to correspond to novel compounds, nimbolide B (**1**) and nimbic acid B (**2**). Nimbolide B inhibited the growth of cress and barnyard grass at concentrations greater than 0.1‒3.0 μM. Nimbic acid B inhibited the growth of cress and barnyard grass at concentrations greater than 0.3–1.0 μM. These results suggest that nimbolide B and nimbic acid B may contribute to the allelopathic effects caused by neem leaves.

## 1. Introduction

Neem (*Azadirachta indica*) belongs to the *Meliaceas* family and grows in the Indian subcontinent and Southeast Asia. The species has long history as a traditional ingredient for household remedies. Oil extracts from the seeds have been used for soaps and cosmetics, and twigs of the plants has been used as tooth-brushes. Farmers also used traditionally various parts of the plants to control insect pests in stored crops and for livestock diseases [1,2,3]. Its insecticidal properties and low toxicity to mammals have particularly attracted scientists in chemistry, pharmacology and agriculture, and many bioactive compounds have been identified in the plant such as nimbin (anti-inflammatory), nimbidin (anti-bacterial, anti-ulcer), nimbidol (anti-tubercular, anti-protozoan), gedunin (anti-malaria, anti-fungal), sodium nimbinate (diuretic, anti-arthritic) and salannin (repellent) [2,4,5,6,7].

It was also reported that litter and extracts of neem plants have strong herbicidal or allelopathic activity [8,9]. However, only a few phenolic compounds have been reported as allelopathic compounds of the plants [4,10]. Phenolic compounds are universal in many plant species and such allelopathic activity of neem cannot be distinguished from that of other plant species only by the phenolic compounds. Therefore, there may be another allelopathic substances in neem. Allelopathic substances have potential as either herbicides or templates for new synthetic herbicide classes [11,12,13,14,15]. Natural compounds are considered to be more environmentally benign than most synthetic herbicides [14,15]. Neem is considered a promising source of herbicides or templates for new synthetic herbicide classes because of its low toxicity to mammals. The objective of this study was the investigation of the possible allelopathic properties and allelopathic active substances of neem.

## 2. Results and Discussion

### 2.1. Allelopathic Activity of the Extracts of Neem Leaves

**Figure 1:**
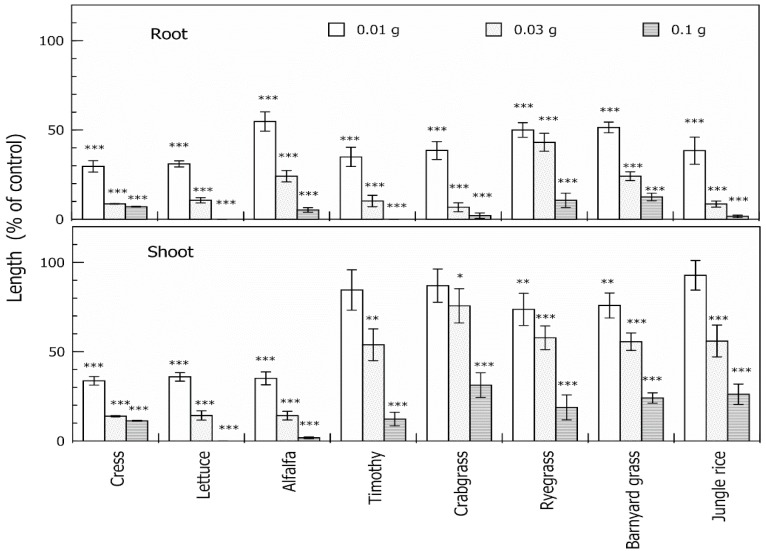
Effects of aqueous methanol extracts of neem leaves on root and shoot growth of cress, lettuce, alfalfa, timothy, crabgrass, ryegrass, barnyard grass and jungle rice*.* Concentrations of tested samples corresponded to the extract obtained from 0.01, 0.03 and 0.1 g dry weight of neem leaves per mL. Means ± SE from four independent experiments with 10 plants for each determination are shown. Asterisk indicates significant difference between control and treatment: * *p* < 0.05, ** *p* < 0.01, *** *p* < 0.001.

Aqueous methanol extracts of neem leaves inhibited root and shoot growth of all test plant species, and increasing the extract concentration resulted in an increase in the inhibition (Figure 1). The extract obtained from 0.1 g dry weight of neem leaves inhibited the root growth of cress, lettuce, alfalfa, timothy, crabgrass, ryegrass, barnyard grass and jungle rice to 7.0%, 0%, 5.2%, 0%, 2.0%, 10.6%, 0%, and 1.6% of control root growth, respectively, and inhibited the shoot growth of cress, lettuce, alfalfa, timothy, crabgrass, ryegrass, barnyard grass and jungle rice to 11.3%, 0%, 1.8%, 12.3%, 31.3%, 18.8%, 24.1% and 26.2% of the control shoot growth, respectively. Therefore, the extracts of neem leaves had inhibitory effects on both dicotyledonous (cress, lettuce and alfalfa) and monocotyledonous plants, including weed species (timothy, crabgrass*,* ryegrass, barnyard grass and jungle rice). These results suggest that neem leaves have allelopathic properties and thus, contain allelopathic substances. In addition, the sensitivity of the roots of monocotyledonous plant species to the extracts was higher than that of those shoots (Figure 1).

The negative impacts of commercial herbicide use on the environment make it desirable to diversify weed management options [11,16,17]. Some plants provide excellent weed control in intercropping and/or as soil additives [16,18,19]. Plants produce hundreds of secondary metabolites, and some of these compounds show allelopathic activity such as growth inhibitory effects on other plants [11,12,14,15,20]. Thus, allelopathy is one strategy to reduced commercial herbicide dependency in practical weed control programs. Therefore, neem leaves may be candidate for soil additive materials to control weeds in sustainable agriculture practices.

### 2.2. Isolation and Identification of Allelopathic Active Substances

**Figure 2:**
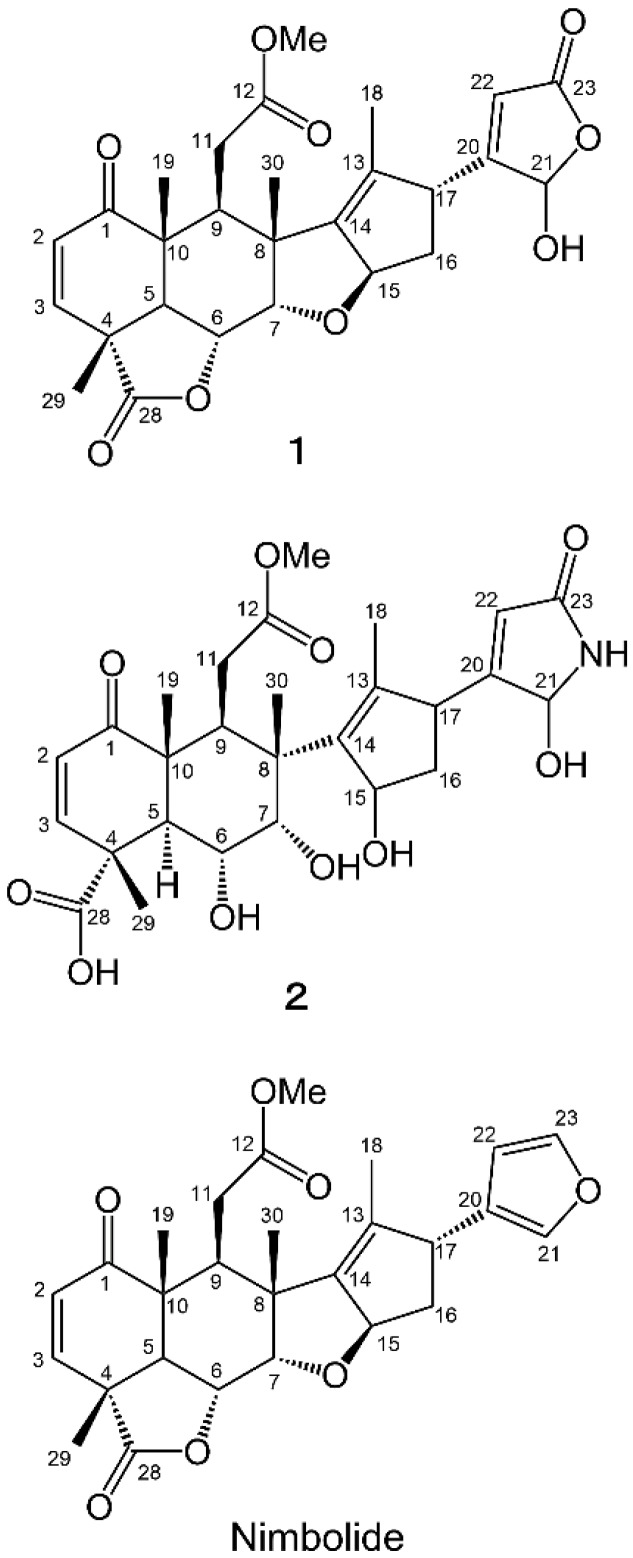
Chemical structures of compounds **1** and **2**.

**Figure 3:**
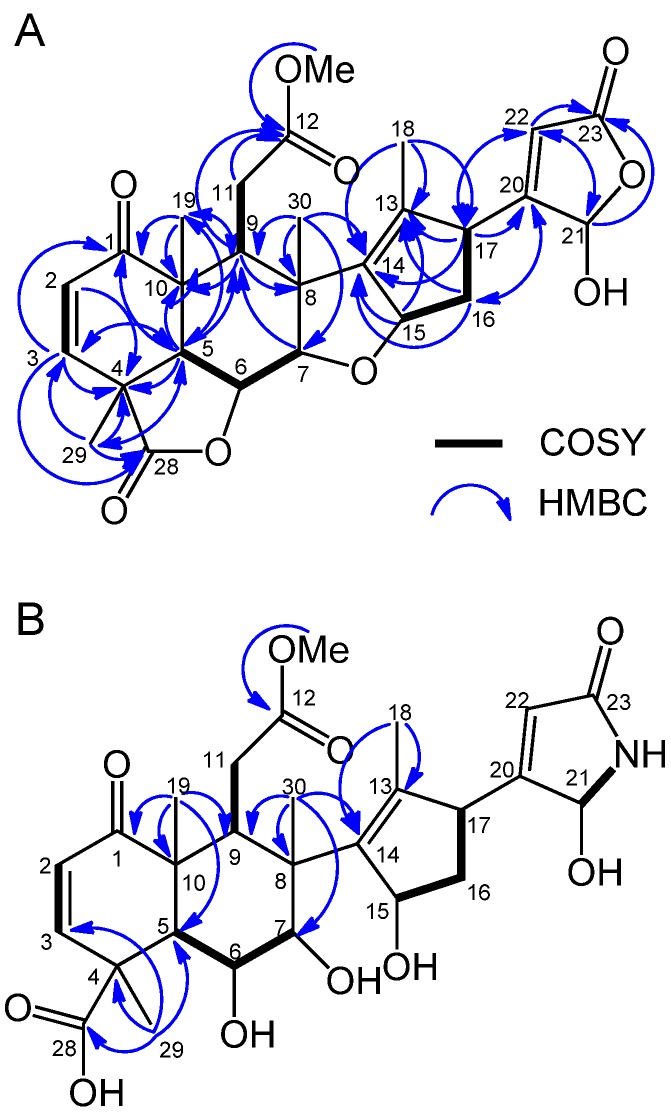
COSY and HMBC correlations of compound **1** (**A**) and **2** (**B**).

**Figure 4:**
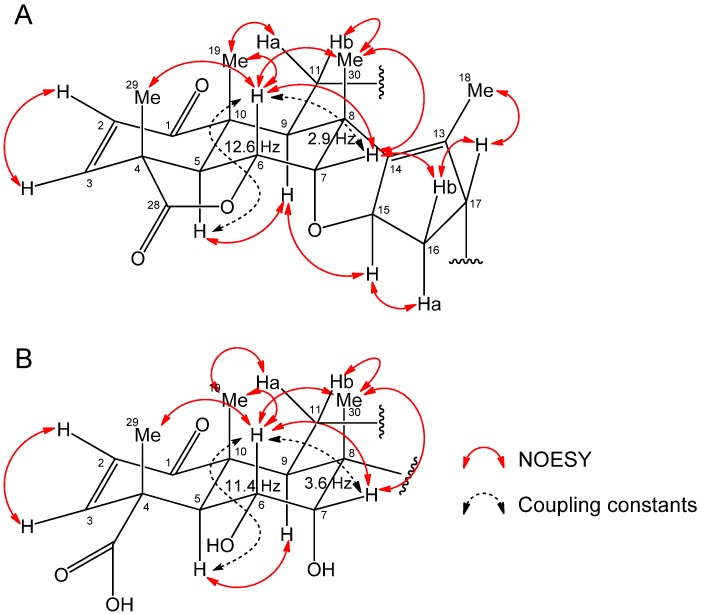
NOESY correlations of compound **1** (**A**) and **2** (**B**).

The aqueous methanol extract was separated on a silica gel column and the biological activity of the fractions was determined. The most active fraction was further purified by Sephadex LH-20, reverse-phase C_18_ Sep-Pak cartridges and HPLC with monitoring the inhibitory activity by cress bioassay and two active compound **1** and **2** were isolated. Compound **1** has a molecular formula of C_27_H_30_O_9_ as suggested by HRESIMS at *m/z* 499.1969 [M+H]^+^ (calcd for C_27_H_31_O_9_, 499.1968) and 497.1809 [M−H]^−^ (calcd for C_27_H_29_O_9_, 497.1812). Compound 1 exhibited IR (neat) absorption bands at 3395 (br, hydroxyl), 1780 (γ-lactone), 1762 (α,β-unsaturated-γ-lactone), 1734 (carbomethoxyl), and 1676 (cyclohexenone) cm^−1^. NMR spectral data of compound **1** (Table 1) showed a basic structure similar to that of nimbolide (Figure 2), which is a known compound isolated from neem [21,22,23]. Therefore, the structure of compound **1** was elucidated based on a comparison of their spectroscopic data to those of nimbolide. An NMR study (^1^H and ^13^C-NMR, COSY, and HMQC) and a consideration of the molecular formula indicated that compound **1** contained five methyl groups, including one methoxy, two methylenes, 10 methines including four oxymethines, and 10 quaternary carbons. A detailed analysis of the COSY and HMQC spectra of compound **1** in CDCl_3_ allowed us to elucidate the following four partial structures: C2-C3, C5-C7, C9-C11, and C15-C17. The connectivity of these fragments was elucidated based on the HMBC techniques. As shown in Figure 3A, HMBC analyses indicated that the planar structure of compound **1** was identical to that of nimbolide, except for the furan moiety. Instead, HMBC correlations from H21 (δ_H_ 5.77) to C22 (δ_C_ 120.3) and carbonyl carbon C23 (δ_C_ 170.2), and from H22 (δ_H_ 5.92) to C21 (δ_C_ 97.4) and C23 established the α,β-unsaturated-γ-lactone ring structure. The NOESY spectrum of compound **1** and the coupling constants demonstrated that the relative stereostructure of the A-E ring system (C1-C17) of compound **1** was also the same as that of nimbolide (Figure 4A). The large magnitude of *J*_5,6_ (12.5 Hz) indicated that the protons H5 and H6 had an axial orientation. The NOESY correlations of H5/H9, H6/H7, H6/H19, H6/H29, H6/H30, and H7/H30 revealed that the 6,6-bicyclic ring system was present in a half-chair-chair conformation. The NOESY correlations of H9/H15, H15/H16b, H7/H16b, H16b/H17, and H17/H18 confirmed the relative steroschemistries at C8, C15 and C17. Thus, the structure of compound **1** was determined to be a novel compound, nimbolide B (Figure 2). Stereochemistry of the C21 position remains to be determined.

**Table 1:** NMR data for compounds **1** and **2** in CDCl_3_.

	Compound **1**		Compound **2**	
Position	δ_H_/ppm (mult., *J* in Hz) ^a^	δ_C_/ppm ^b^	δ_H_/ppm (mult., *J* in Hz) ^a^	δ_C_/ppm ^c^
1		200.8		202.2
2	5.91 (d, 9.9)	130.8	5.85 (d, 10.1)	126.0
3	7.28 (d, 9.9)	150.0	6.44 (d, 10.1)	148.5
4		43.7		43.0
5	3.08 (d, 12.5)	47.8	3.31 (d, 11.4)	47.9
6	4.60 (dd, 2.9, 12.5)	73.2	3.92 (m)	n.d. ^d^
7	4.32 (d, 3.4)	83.1	4.07 (d, 3.6)	87.9
8		50.6		46.9
9	2.60 (m)	41.4	2.61 (m)	39.1
10		45.4		43.3
11a	2.30 (m)	32.8	2.12 (dd, 2.5, 16.5)	35.0
11b	3.18 (m)		2.83 (dd, 6.4, 16.5)	
12		175.0		175.8
13		132.5		130.9
14		148.6		150.4
15	5.60 (dd, 7.1, 7.1)	88.8	5.66 (m)	87.5
16a	2.16 (m)	38.9	2.10 (dd, 2.0, 12.5)	38.4
16b	2.39 (m)		2.41 (dd, 6.7, 12.5)	
17	3.81 (d, 9.4)	53.0	3.86 (m)	57.6
18	1.74 (s, 3H)	13.1	1.73 (s, 3H)	13.0
19	1.23 (s, 3H)	14.9	1.23 (s, 3H)	15.7
20		168.4		n.d. ^d^
21	5.77 (s)	97.4	5.74 (d, 7.6)	97.1
22	5.92 (s)	120.3	5.95 (s)	120.0
23		170.2		n.d. ^d^
28		174.7		174.8
29	1.48 (s, 3H)	18.7	1.60 (s, 3H)	17.0
30	1.35 (s, 3H)	17.1	1.28 (s, 3H)	17.1
12OCH_3_	3.76 (s, 3H)	52.7	3.80 (s, 3H)	52.2
21NH			5.56 (d, 7.6)	

^a^ Recorded at 400 MHz. ^b^ Recorded at 100 MHz. ^c^ Based on HMQC and HMBC spectra. ^d^ Not detected.

Compound **2** has a molecular formula of C_27_H_35_NO_10_ as suggested by HRESIMS at *m/z* 534.2364 [M+H]^+^ (calcd for C_27_H_36_NO_10_, 534.2339) and 532.2198 [M−H]^−^ (calcd for C_27_H_34_NO_10_, 532.2183). Compound **2** exhibited IR (neat) absorption bands at 3402 (br, hydroxyl), 1761(carbomethoxyl), 1732 (carboxylic acid), 1715 (α,β-unsaturated-γ-lactam), and 1682 (cyclohexenone) cm^−1^. NMR spectral data of compound **2** (Table 1) also showed similarity in the structure with nimbolide B and nimbolide. A detailed analysis of the ^1^H-NMR, COSY and HMQC spectra of compound **2** in CDCl_3_ allowed us to elucidate the following five partial structures: C2-C3, C5-C7, C9-C11, C15-C17, and C21-NH. ^1^H-NMR signal of 21NH (β_H_ 5.56) was confirmed to be diminished with the addition of D_2_O. The connectivity of these fragments was elucidated based on the HMBC analysis as shown in Figure 3B. From the COSY correlation of H21/NH, and the observation of the different signals in NMR spectra of compound **2** from those of nimbolide B around the α,β-unsaturated-γ-lactone moiety, compound **2** was elucidated to possess an α,β-unsaturated-γ-lactam ring structure. In addition, the lack of the lactone carbonyl absorption from the IR spectrum and the estimated molecular formula suggested the structure did not include lactone and ether rings. The NOESY spectrum of compound **2** and the coupling constants (*J*_5,6_ = 11.4 Hz, *J*_6,7_ = 3.6 Hz,) demonstrated that the relative stereostructure of the 6,6-bicyclic ring system (C1-C10) of compound **2** was the same as that of nimbolide B (Figure 4B). Thus, the structure of compound **2** was also determined to be a novel compound, nimbic acid B (Figure 2). Stereochemistry of the C15, C17, and C21 positions remain to be determined.

### 2.3. Biological Activity

**Figure 5:**
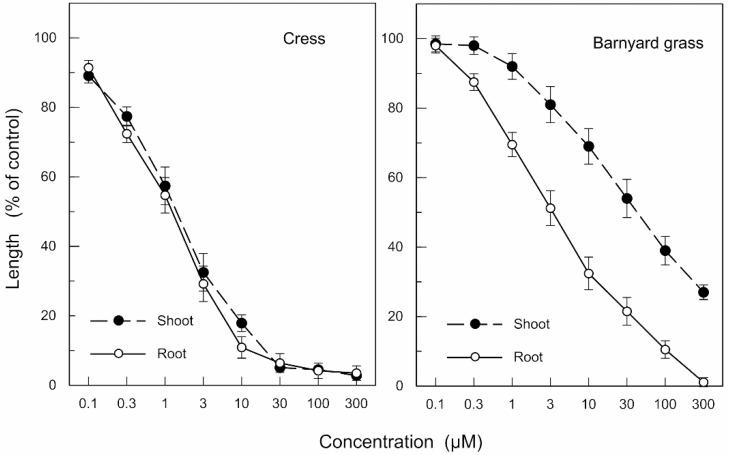
Effects of nimbolide B on the root and shoot growth of cress and barnyard grass. Means ± SE from 4 independent experiments with 10 plants for each determination are shown.

The biological activities of nimbolide B and nimbic acid B isolated from neem were determined with cress and barnyard grass. Nimbolide B inhibited the growth of cress roots and shoots at concentrations greater than 0.1 μM, and the growth of barnyard grass roots and shoots at concentrations greater than 0.3 and 3 μM, respectively (Figure 5). The inhibition was increased with increased concentration of nimbolide B. The concentrations required for 50% growth inhibition of the cress roots and shoots in the assay (defined as I_50_), as determined by a logistic regression analysis, were 1.2 and 1.4 μM, respectively, and I_50_ of the barnyard grass roots and shoots were 3.7 and 39 μM, respectively.

**Figure 6:**
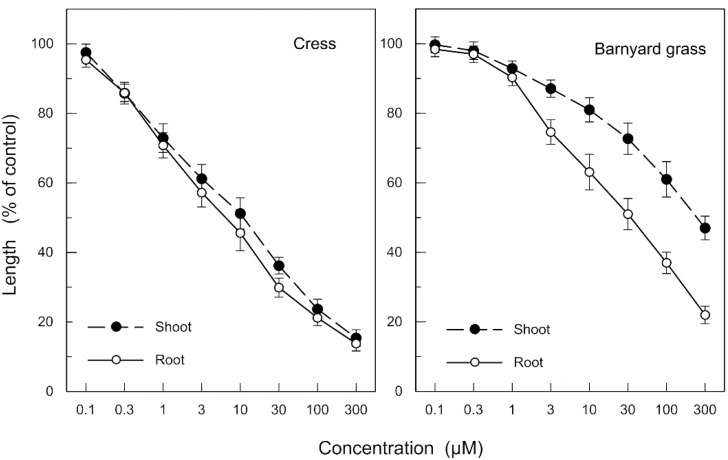
Effects of nimbic acid B on the root and shoot growth of cress and barnyard grass. Means ± SE from 4 independent experiments with 10 plants for each determination are shown.

Nimbic acid B inhibited the growth of cress roots and shoots at concentrations greater than 0.3 μM, and the growth of barnyard grass roots and shoots at concentrations greater than 1.0 μM (Figure 6). I_50_ of the cress roots and shoots were 5.7 and 9.4 μM, respectively, and I_50_ of the barnyard grass roots and shoots were 29 and 210 μM, respectively. Comparing the I_50_ values, the growth inhibitory activity of nimbolide B was 4.8–7.8-fold greater than that of nimbic acid B. The effectiveness of nimbolide B on the cress roots and shoots was 3.1- and 28-fold greater than that of barnyard grass roots and shoots, and the effectiveness of nimbic acid B on the cress roots and shoots was 5.1- and 22-fold greater than that of barnyard grass roots and shoots, which was consistent with results of Figure 1.

As already described in the Introduction, neem extracts and litter were reported to have strong allelopathic activity [8,9], and several phenolic compounds were identified as allelopathic compounds [4,10]. However, many plant species contain phenolic compounds [20] and such strong alleopathic activity of neem cannot be accounted for by only phenolic compounds. Nimbolide B and nimbic acid B, novel compounds in neem, showed growth inhibitory activity at concentrations greater than 0.1–1.0 μM. Therefore, nimbolide B and nimbic acid B may contribute to the allelopathic activity of neem.

## 3. Experimental

### 3.1. Plant Materials

Leaves of neem (*Azadirachta indica*. A. Juss) were collected from the Bangladesh Agricultural University campus, Mymensingh, Bangladesh in February 2010. The leaves were sun-dried and stored at 4 °C until extraction. Seeds of cress (*Lepidium sativum* L.), lettuce (*Lactuca sativa* L.), alfalfa (*Medicago sativa* L.) and timothy (*Phleum pratense* L.) were chosen as test plants for bioassay owing to their known seedling growth behavior. Weed species, ryegrass (*Lolium multiflorum* L.), crabgrass (*Digitaria sanguinalis* L.), barnyard grass (*Echinochloa crus-galli* [L.] Beauv.) and jungle rice (*Echinochloa colonum* [L.] Link.) were chosen for the bioassays.

### 3.2. Extraction and Bioassay

Dried neem leaves (50 g dry weight) were extracted with 80% (*v/v*) aqueous methanol (500 mL) for two days. After filtration using filter paper (No. 2; Toyo, Tokyo, Japan), the residue was extracted again with 500 mL of methanol for one day and filtered. Then the two filtrates were combined. An aliquot of the extract (final assay concentration was 0.01, 0.03 and 0.1 g dry weight neem leaves equivalent extract mL^−1^) was evaporated to dryness, dissolved in methanol (0.2 mL) and added to a sheet of filter paper (No. 2; Toyo, Tokyo) in a 3-cm Petri dish. Methanol was evaporated in a fume hood. Then, the filter paper in the Petri dishes was moistened with 0.8 mL of a 0.05% (*v/v*) aqueous solution of Tween 20. Ten seedlings of cress, lettuce, alfalfa, timothy, crabgrass, ryegrass, barnyard grass or jungle rice were placed on the filter paper in Petri dishes after germination in the darkness at 25 °C for 16 h (cress, lettuce), 24 h (alfalfa), 36 h (timothy) and 48 h (crabgrass, ryegrass, barnyard grass, jungle rice). The length of roots and shoots of these seedlings was measured after 48 h of incubation in the darkness at 25 °C, and the percentage length of the seedlings was determined by reference to the elongation of control seedlings. For control treatment, methanol (0.2 mL) was added to the filter paper in the Petri dish and evaporated as described above. Controls were treated exactly as described above, with the exception that 0.2 mL 80% methanol was used instead of the extract. The bioassay was repeated four times using a completely randomized design with 10 test plants for each determination. Significant differences between treated and control plants were examined by Welch’s *t*-test for each test plant species.

### 3.3. Purification of Active Substances

Neem leaves (500 g dry weight) were extracted as described above and the extract was concentrated at 40 °C *in vacuo* to produce an aqueous residue. The aqueous residue was adjusted to pH 7.0 with 1 M phosphate buffer, and partitioned three times against an equal volume of ethyl acetate. The ethyl acetate fraction was evaporated to dryness and separated on a column of silica gel (100 g, silica gel 60, 70–230 mesh; Merck), eluted stepwise with *n*-hexane containing increasing amounts of ethyl acetate (10% per step, *v/v*; 100 mL per step) and methanol (200 mL). The biological activity of the fractions was determined using a cress bioassay as described above, and activity was found in a fraction obtained by elution with 80% ethyl acetate in *n*-hexane fraction. The active fraction was evaporated and purified using a column of Sephadex LH-20 (100 g, Amersham Pharmacia Biotech, Buckinghamshire, UK), and eluted with 20%, 40%, 60% and 80% (*v/v*) aqueous methanol (100 mL per step) and methanol (200 mL). The active fraction was eluted with 40% aqueous methanol and evaporated to dryness. The residue was dissolved in 20% (*v/v*) aqueous methanol and loaded onto reverse-phase C_18_ Sep-Pak cartridges (Waters). The cartridge was eluted with 20%, 40%, 60%, 80% (*v/v*) aqueous methanol (15 mL per step) and methanol (30 mL per step). The active fraction was eluted by 40% aqueous methanol and evaporated to dryness. The residue was finally purified by reverse-phase HPLC (10 mm i.d. × 50 cm, ODS AQ-325; YMC Ltd., Kyoto, Japan) with a flow rate of 1.5 mL/min with 50% aqueous methanol, and detected at 220 nm. Inhibitory activity was found in peak fractions eluted between 59–62 (compound **2**, 7.5 mg) and 92–95 min (compound **1**, 1.5 mg). These active substances were characterized by high-resolution ESI mass, IR, ^1^H-NMR (400 MHz, CDCl_3_, TMS as internal standard), HMBC, NOESY and ^13^C-NMR spectra (100 MHz, CDCl_3_, TMS as internal standard) and optical rotation.

### 3.4. Spectral Data

Compound **1**. 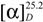 = +133.5 ° (*c* 0.3, CHCl_3_); HRESIMS *m/z* 499.1969 [M+H]^+^ (calcd for C_27_H_31_O_9_, 499.1968, Δ = +0.1 mmu), 497.1809 [M−H]^−^ (calcd for C_27_H_29_O_9_, 497.1812, Δ = −0.3 mmu); IR (neat) 3395 (br), 2926, 2857, 1780, 1762, 1734, 1676, 1456, 1293, 1240, 1128 cm^−1^; NMR data (Table 1).

Compound **2**. 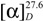 = +43.0 ° (*c* 0.03, CHCl_3_); HRESIMS *m/z* 534.2364 [M+H]^+^ (calcd for C_27_H_36_NO_10_, 534.2339, Δ = +2.5 mmu), 532.2198 [M−H]^−^ (calcd for C_27_H_34_NO_10_, 532.2183, Δ = +1.5 mmu); IR (neat) 3402 (br), 2955, 2924, 2853, 1761, 1732, 1715, 1682, 1437, 1265, 1144 cm^−1^; NMR data (Table 1).

### 3.5. Bioassay of the Isolated Compounds

Compounds **1** or **2** were dissolved in methanol (0.2 mL), added to a sheet of filter paper (No. 2) in a 3-cm Petri dish, and the methanol was evaporated in a fume hood. The filter paper in the Petri dish was moistened with 0.8 mL of 0.05% (*v/v*) aqueous Tween 20. Ten seedlings of cress or barnyard grass were placed on the filter paper in Petri dishes after germination in the darkness at 25 °C for 24 and 72 h, respectively. The length of roots and shoots of these seedlings were measured after 48 h of incubation in the darkness at 25 °C, and the percentage length of the seedlings was determined by reference to the elongation of control seedlings as described above.

## 4. Conclusions

Aqueous methanol extracts of neem leaves had allelopathic activity (Figure 1) and two potent growth inhibitory substances (compounds **1** and **2**) causing the allelopathic effect were isolated. The chemical structures of compound **1** and **2** identified them as novel compounds, nimbolide B and nimbic acid B, respectively (Figure 2). These compounds were active at concentrations greater than 0.1–1.0 μM. The I_50_ values of nimbolide B and nimbic acid B were 1.2–39 μM and 5.7–210 μM, respectively, therefore, nimbolide B and nimbic acid B may contribute to the allelopathic activity of neem. Allelopathy is one strategy to reduce the dependency on commercial herbicides in practical weed control programs. Neem leaves may be candidate for soil additive materials to control weeds in sustainable agriculture.

## Supplementary Materials

Supplementary materials can be accessed at: http://www.mdpi.com/1420-3049/19/6/6929/s1.
