# Supplementary material for: Nimbolide B and Nimbic Acid B, Phytotoxic Substances in Neem Leaves with Allelopathic Activity
Source: Molecules. 2014 May 26;19(6):6929–40. doi: 10.3390/molecules19066929 (PMC6290558; doi:10.3390/molecules19066929)
Supplement: Supplementary file 1 [file molecules-19-06929-s001.pdf]

Supplementary Materials

Figure S1. ^{1}H NMR spectrum of compound **1** (400 MHz, $CDCl_3$).

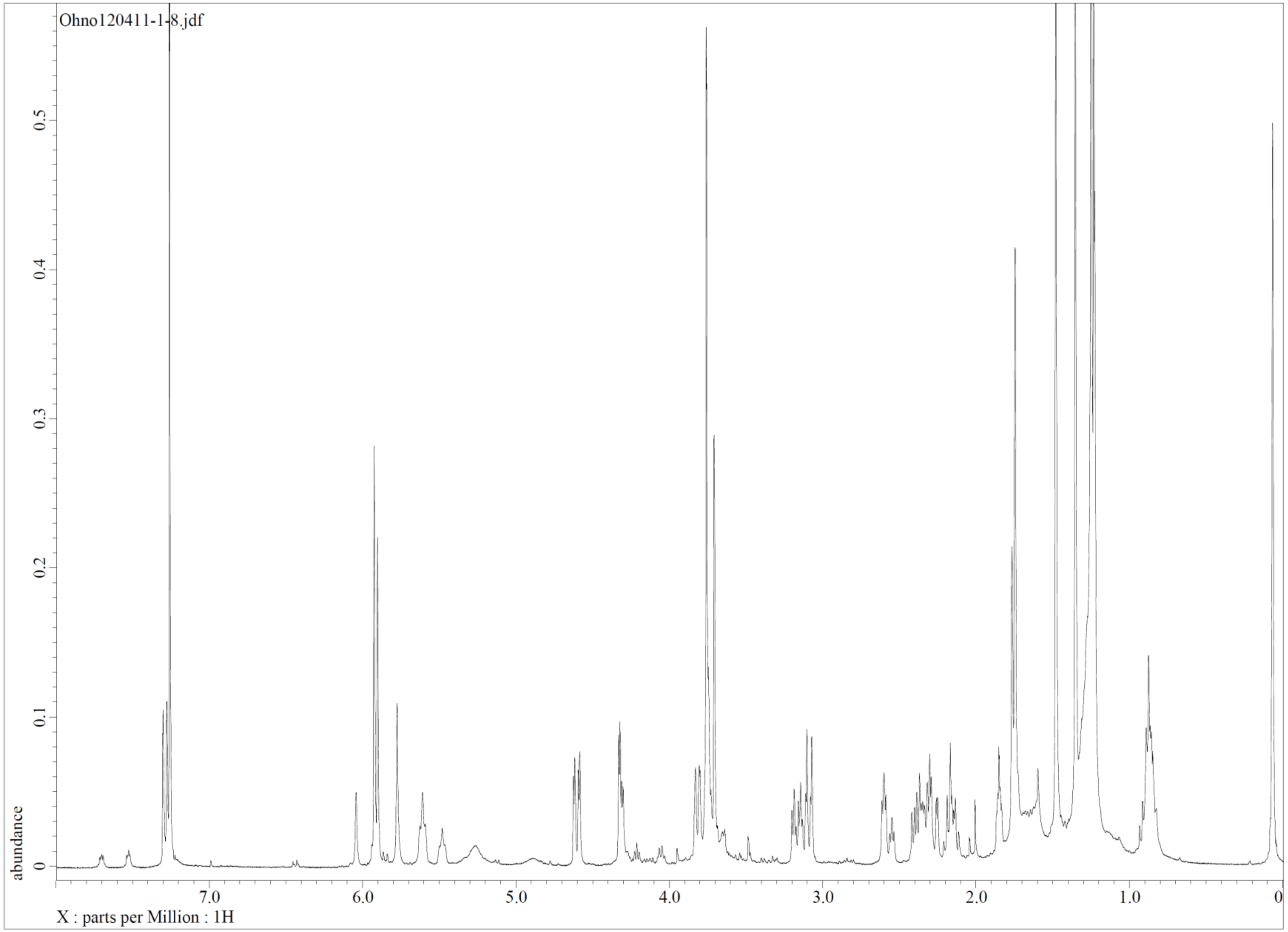

Figure S2. ^{13}C NMR spectrum of compound **1** (100 MHz, $CDCl_3$).

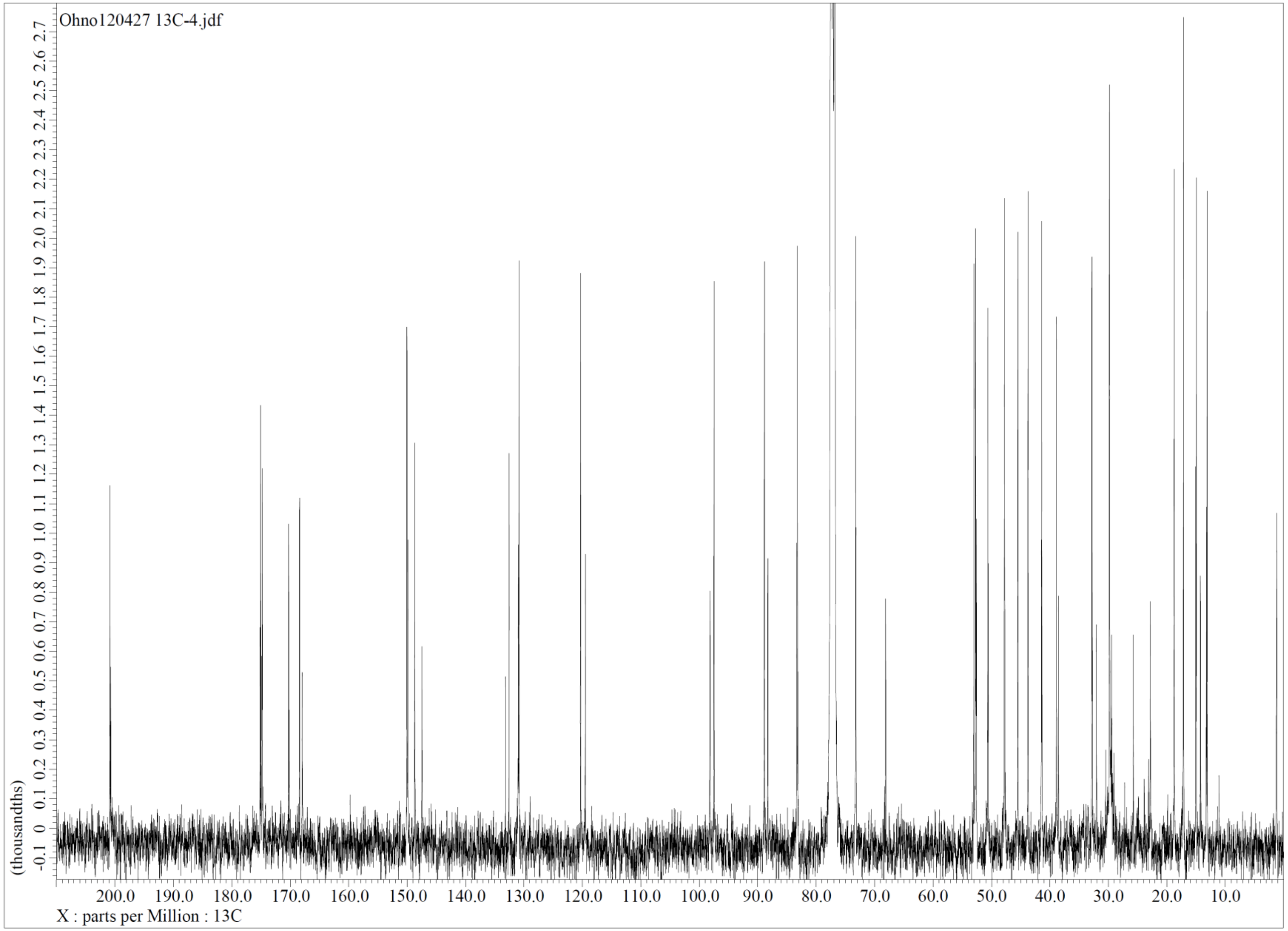

Figure S3. COSY spectrum of compound **1** (400 MHz, $CDCl_3$).

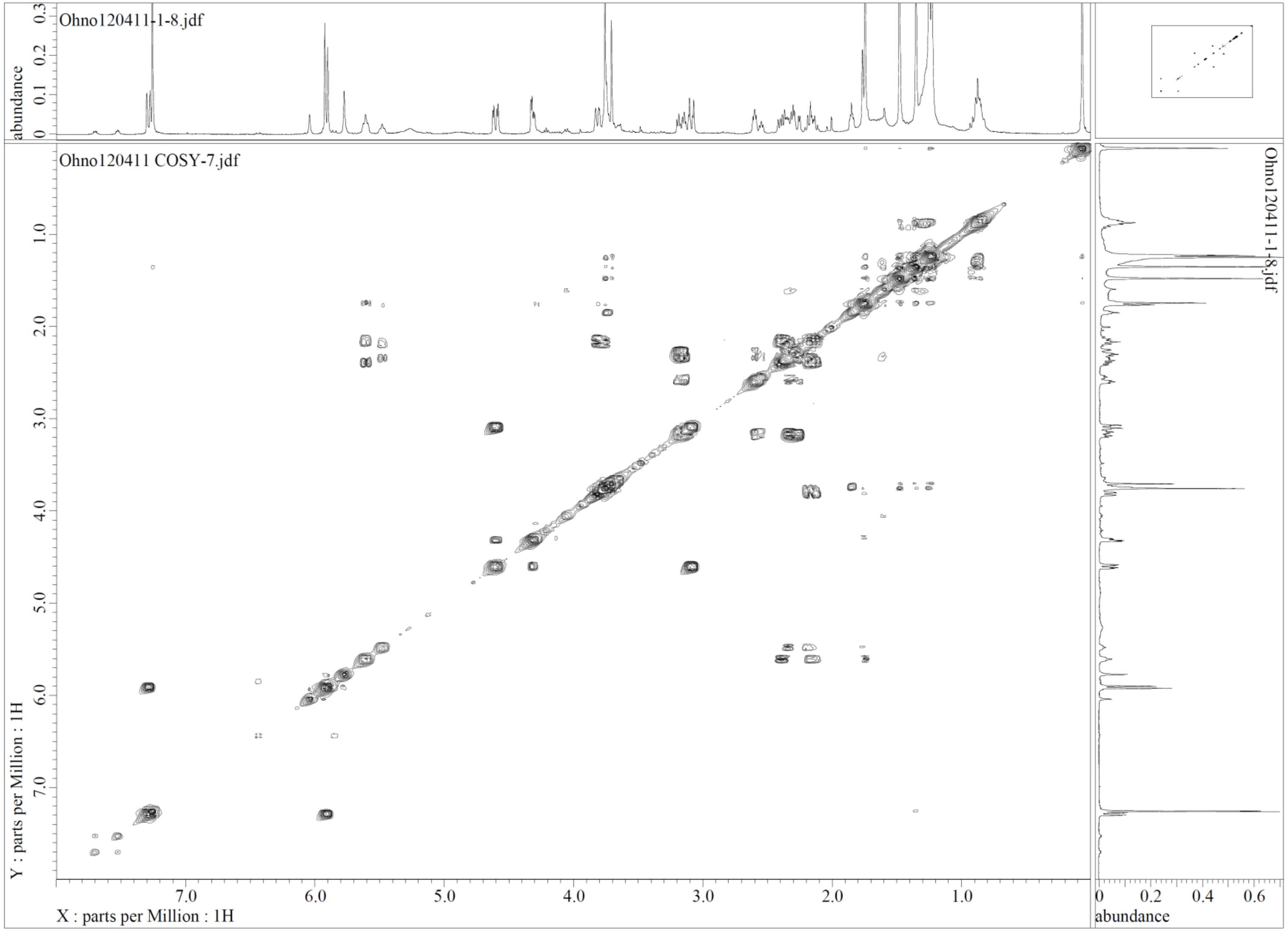

Figure S4. HMQC spectrum of compound **1** (400 MHz, $CDCl_3$).

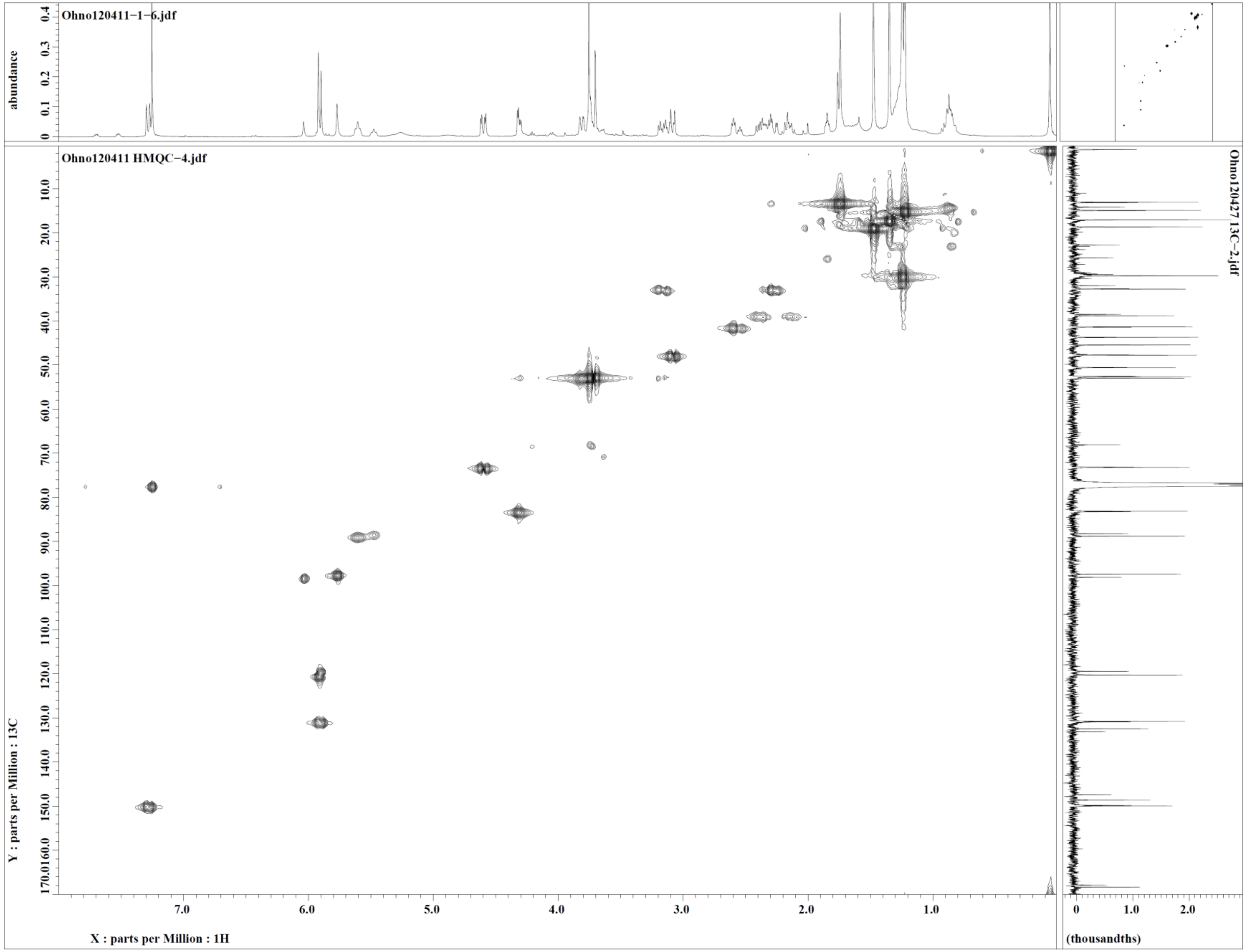

Figure S5. HMBC spectrum of compound **1** (400 MHz, $CDCl_3$).

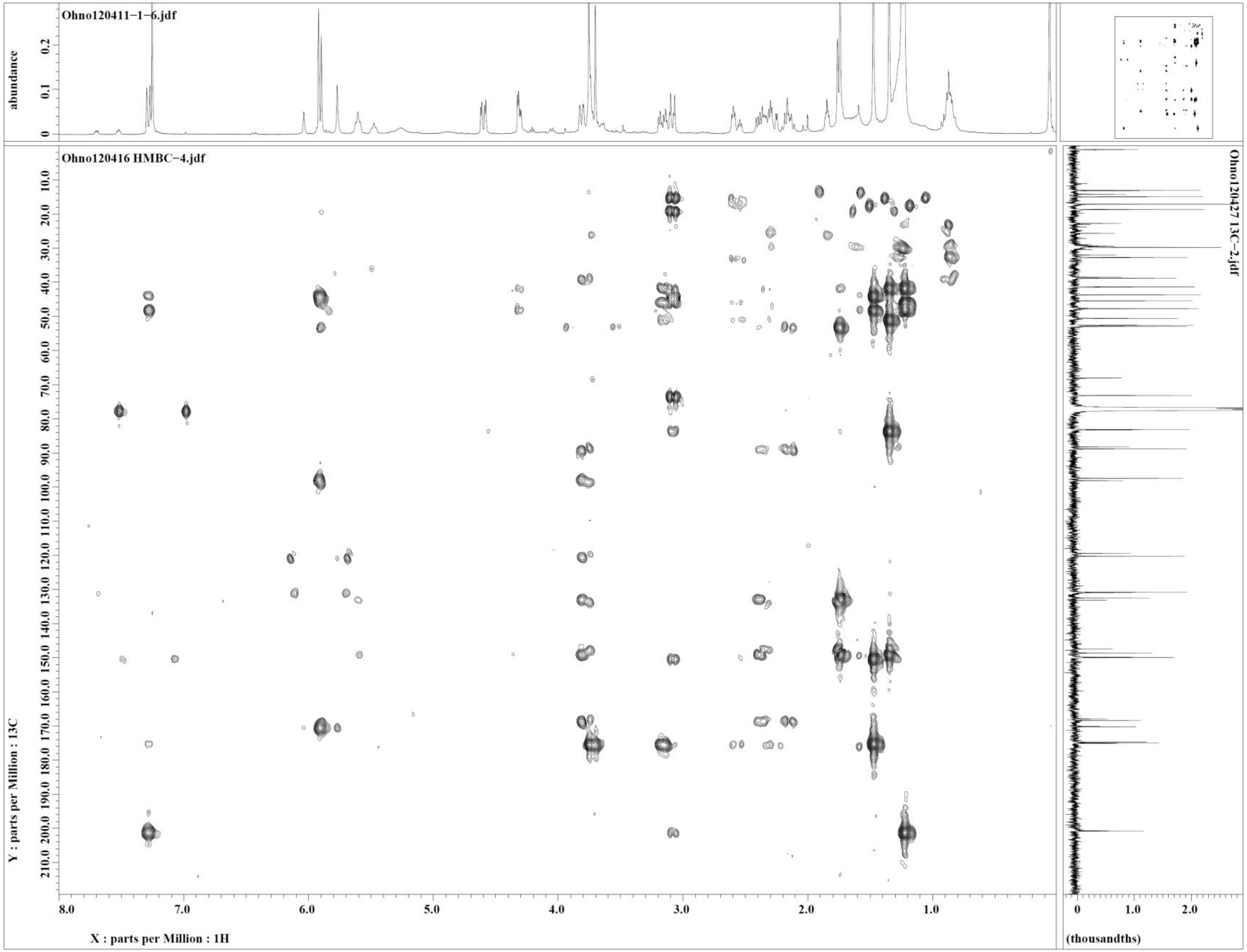

Figure S6. NOESY spectrum of compound **1** (400 MHz, $CDCl_3$).

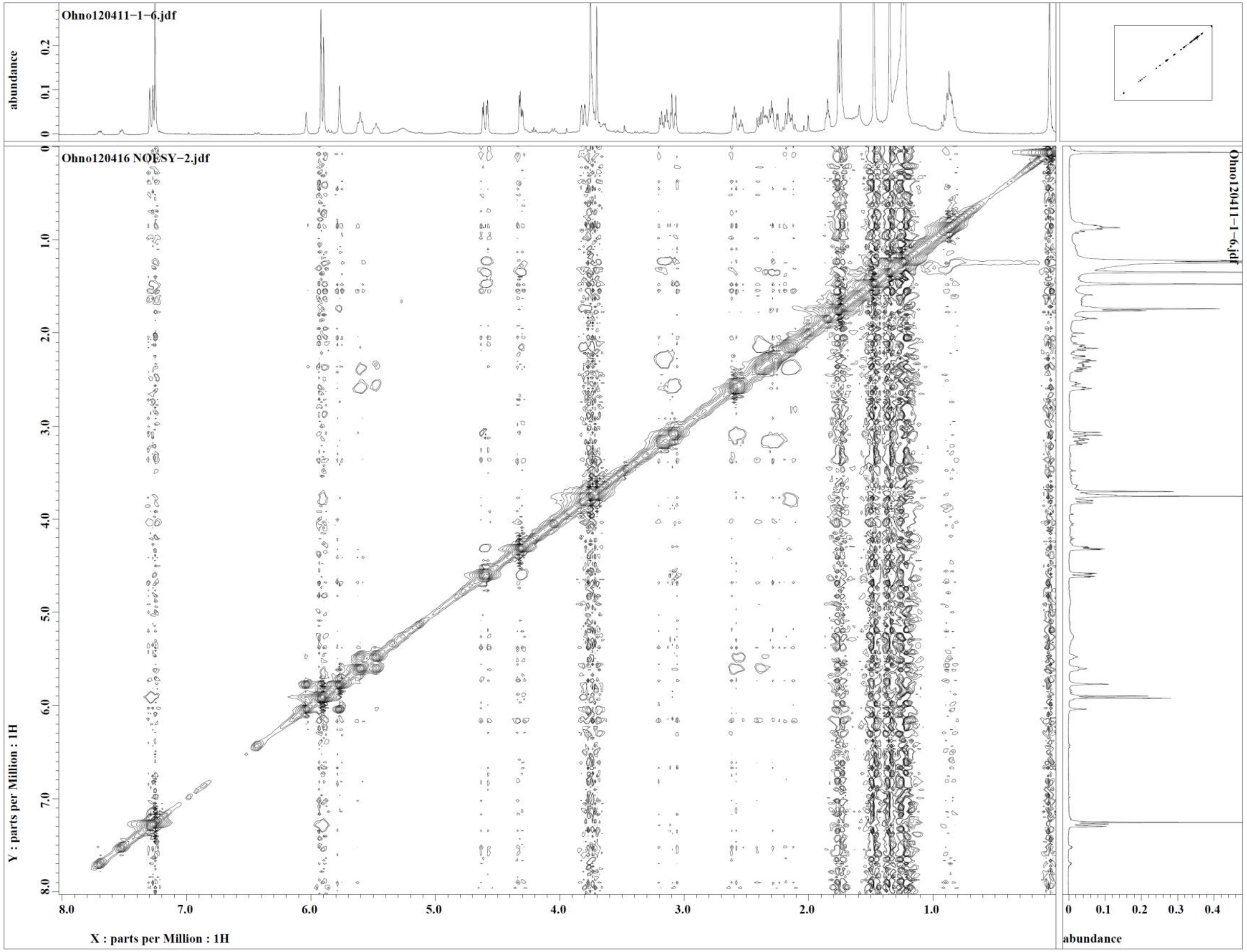

Figure S7. IR spectrum of compound **1** (neat).

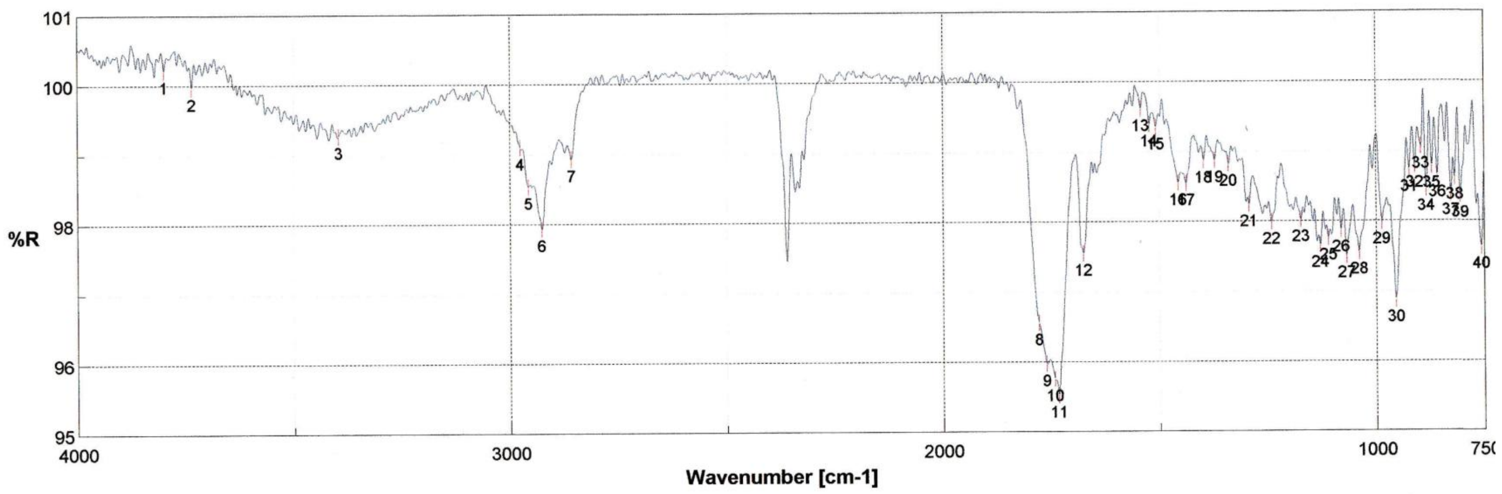

積算回数	64	分解	4 cm-1
ゼロフィリング	ON	アポダイゼーション	Cosine
ゲイン	32	アパーチャー	7.1 mm
スキャンスピード	2 mm/sec		
測定日時	2012/05/02 19:05	更新日時	2012/05/02 19:08
測定者	オペレータ		
ファイル名	Memory#1		
サンプル名	試料名		
コメント	コメント		

No.	cm-1	%R	No.	cm-1	%R	No.	cm-1	%R	No.	cm-1	%R	No.	cm-1	%R
1	3799.08	100.218	2	3734.48	99.9664	3	3395.07	99.2714	4	2975.62	99.1029	5	2957.3	98.5393
6	2926.45	97.9313	7	2857.99	98.9326	8	1779.97	96.5604	9	1761.65	95.9714	10	1743.33	95.762
11	1733.69	95.5271	12	1675.84	97.5557	13	1542.77	99.6154	14	1523.49	99.3995	15	1508.06	99.3507
16	1456.96	98.5577	17	1438.64	98.552	18	1398.14	98.8718	19	1372.1	98.878	20	1340.28	98.8287
21	1293.04	98.2439	22	1240.97	97.9935	23	1173.47	98.026	24	1128.15	97.6598	25	1109.83	97.7636
26	1080.91	97.8759	27	1067.41	97.5056	28	1038.48	97.5579	29	986.411	97.9847	30	953.627	96.8754
31	922.771	98.7337	32	909.272	98.7921	33	895.773	99.0651	34	883.238	98.456	35	869.739	98.7892
36	857.204	98.6539	37	826.348	98.3967	38	816.706	98.6172	39	805.135	98.3615	40	756.923	97.6284

Figure S8. ^{1}H NMR spectrum of compound **2** (400 MHz, $CDCl_3$).

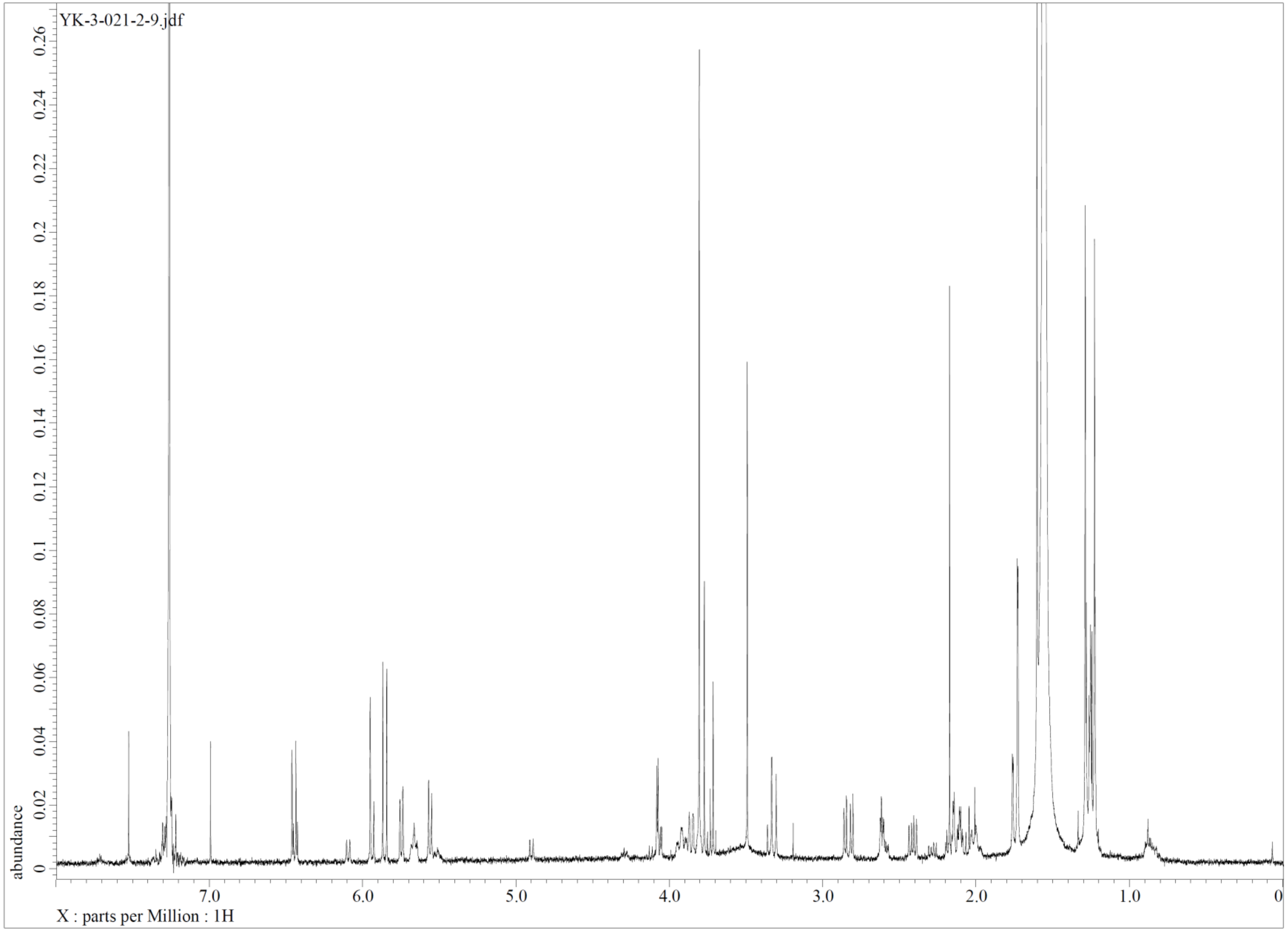

Figure S9. COSY spectrum of compound **2** (400 MHz, $CDCl_3$).

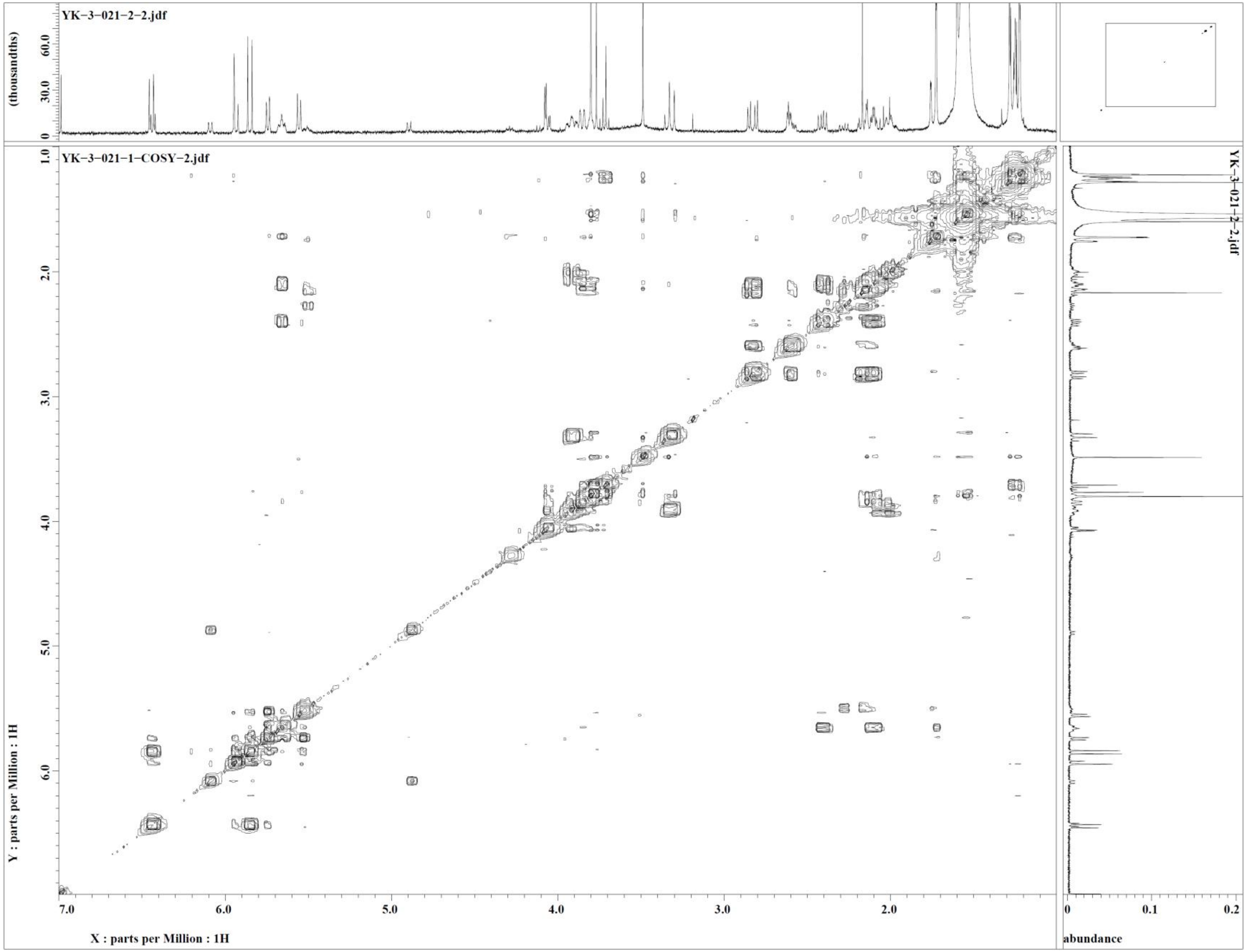

Figure S10. HMQC spectrum of compound **2** (400 MHz, $CDCl_3$).

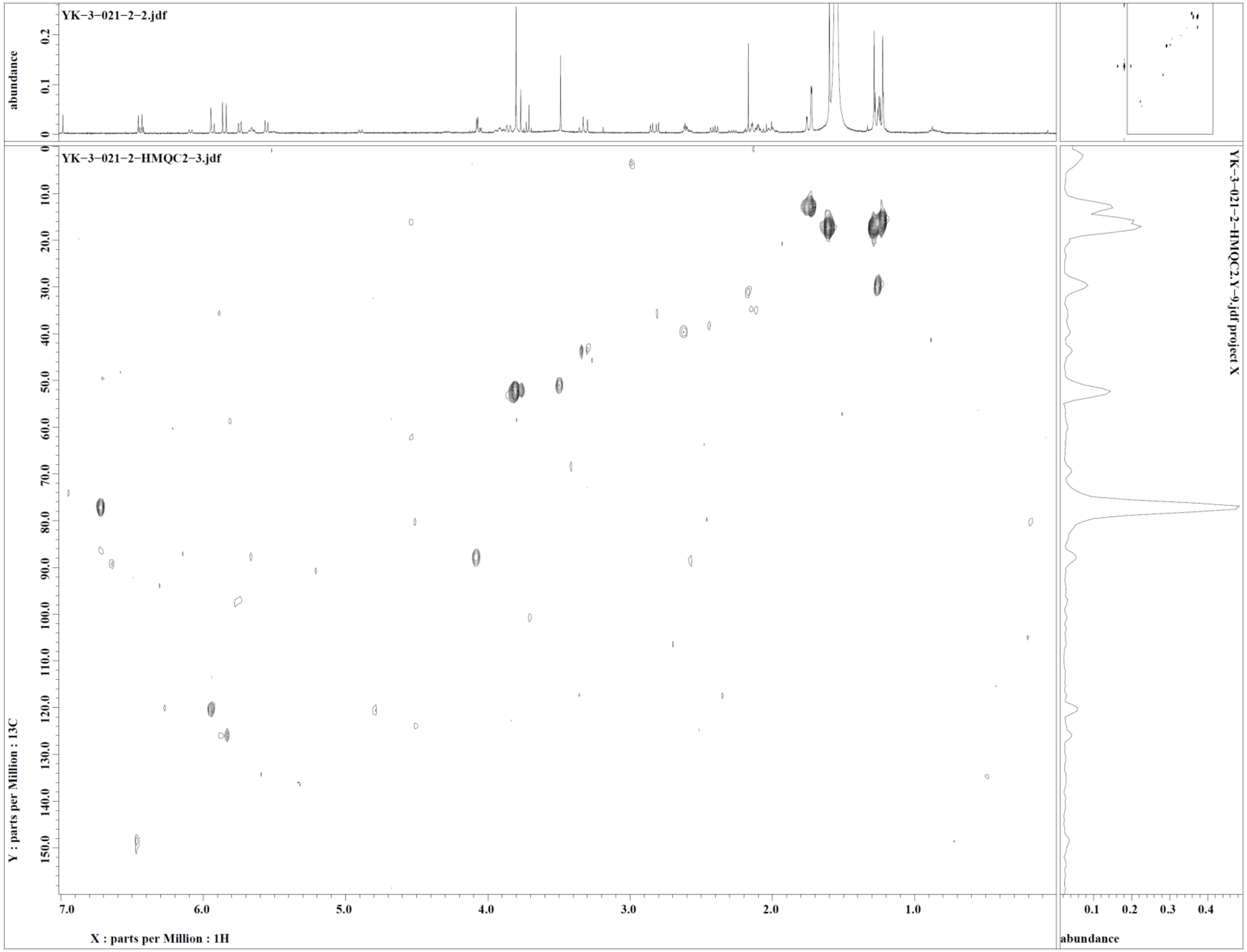

Figure S11. HMBC spectrum of compound **2** (400 MHz, $CDCl_3$).

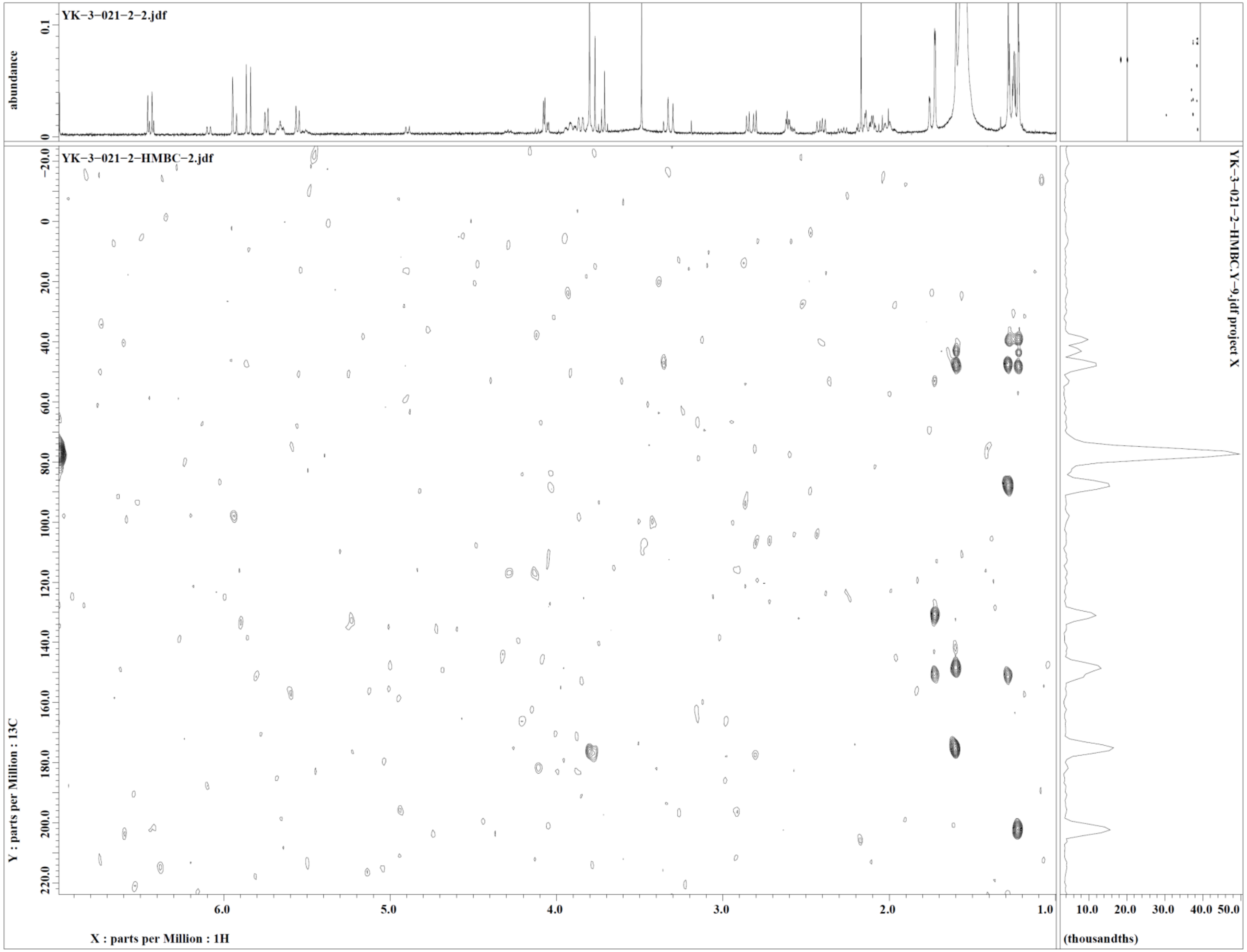

Figure S12. NOESY spectrum of compound **2** (400 MHz, $CDCl_3$).

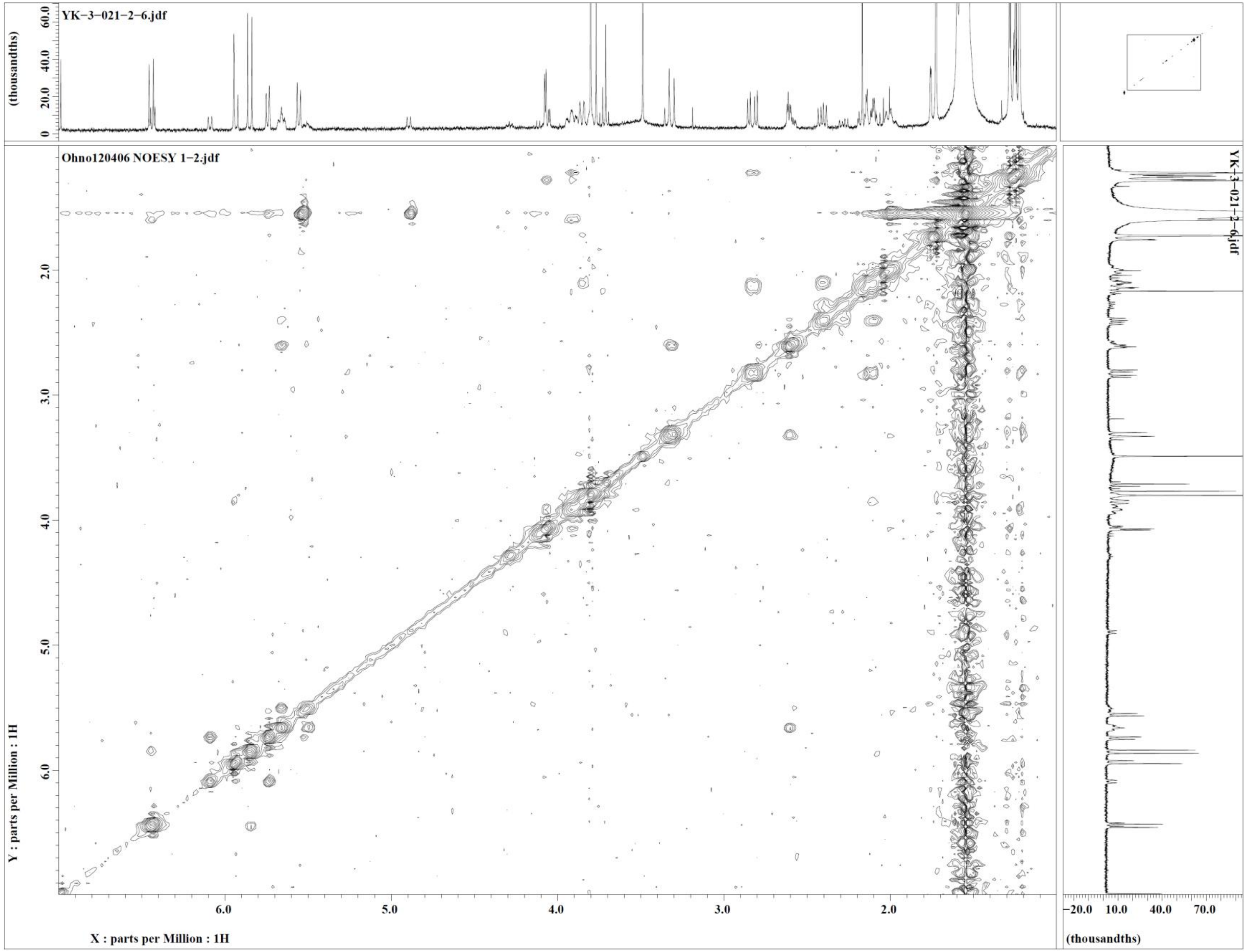

Figure S13. IR spectrum of compound **2** (neat).

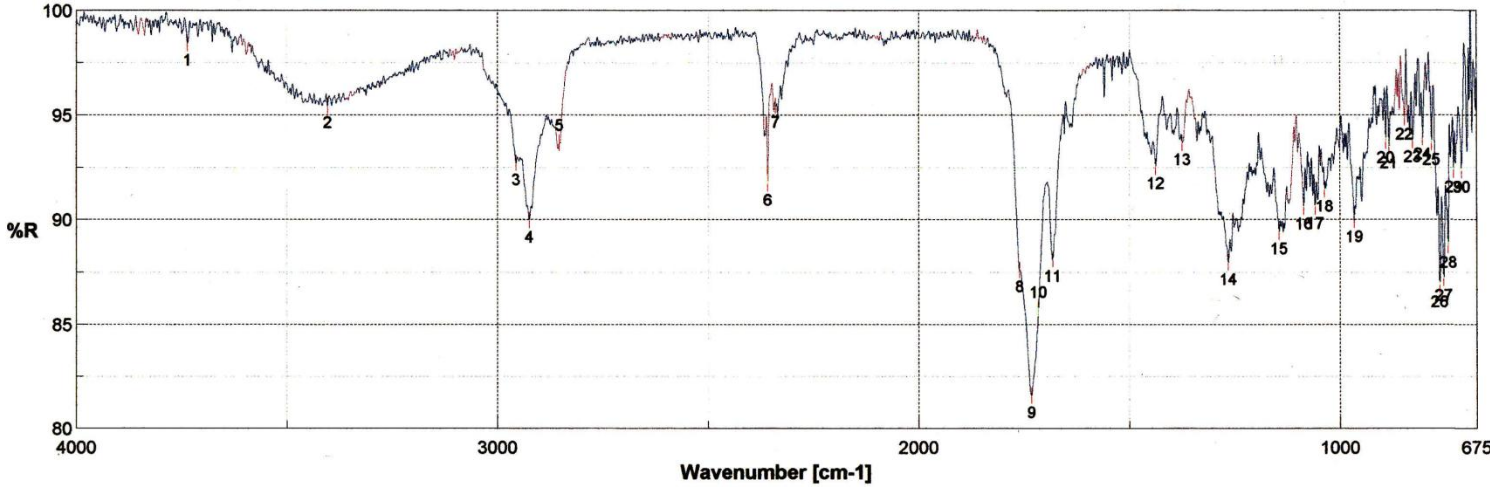

積算回数	32	分解	2 cm-1
ゼロフィリング	ON	アポダイゼーション	Cosine
ゲイン	32	アパーチャー	5 mm
スキャンスピード	2 mm/sec		
測定日時	2012/04/11 12:18	更新日時	2012/04/11 12:32
測定者	オペレータ		
ファイル名	Memory#1		
サンプル名	試料名		
コメント	コメント		

No.	cm-1	%R	No.	cm-1	%R	No.	cm-1	%R	No.	cm-1	%R	No.	cm-1	%R
1	3735.44	98.4273	2	3402.3	95.4418	3	2954.89	92.716	4	2924.04	89.9787	5	2852.68	93.6818
6	2358.03	91.7357	7	2340.19	95.4613	8	1761.17	87.5988	9	1732.25	81.5672	10	1715.85	85.7094
11	1682.11	88.1091	12	1437.19	92.5472	13	1374.51	93.674	14	1265.07	87.9563	15	1143.58	89.4146
16	1085.24	90.6117	17	1058.25	90.63	18	1036.07	91.4532	19	966.162	89.9824	20	889.987	93.7818
21	882.756	93.5339	22	845.633	94.8938	23	826.348	93.8056	24	802.724	93.9536	25	781.511	93.6719
26	762.227	86.8777	27	753.066	87.1809	28	742.46	88.765	29	729.443	92.3681	30	710.64	92.3764
